# Optimal Operative Strategies in Repair of Giant Coronary Artery Aneurysms: Case Presentation

**DOI:** 10.70352/scrj.cr.26-0145

**Published:** 2026-06-23

**Authors:** Takuma Fukunishi, Tomohide Higaki, Tomohisa Sakaue, Hirotsugu Kurobe, Takashi Nishimura, Hironori Izutani

**Affiliations:** Department of Cardiovascular Surgery, Ehime University School of Medicine, Ehime, Japan

**Keywords:** coronary artery aneurysm, coronary artery bypass grafting (CABG), aneurysmorrhaphy, fistula, revascularization

## Abstract

**INTRODUCTION:**

Surgical exclusion of giant coronary artery aneurysms (GCAAs) complicated by coronary-to-left ventricular (LV) fistulae requires a definitive strategy to obliterate the abnormal communication while maintaining myocardial integrity. We report 2 surgical cases focusing on internal orifice closure.

**CASE PRESENTATION:**

In Case 1 (80-year-old male), a calcified left circumflex artery-GCAA was managed by direct internal closure of the aneurysm neck and LV fistula. The procedure focused on isolating the aneurysm from the coronary circulation, with an auxiliary left internal thoracic artery-obtuse marginal (LITA-OM) graft to ensure distal flow. In Case 2 (39-year-old male), the extensive GCAA required a Hemashield patch (GETINGE, Göteborg, Sweden) for internal closure of the left coronary ostium. Simultaneously, the LV fistula was closed from within the aneurysm. Although postoperative LV unloading via percutaneous cardiopulmonary support (PCPS) and Impella (Abiomed, Danvers, MA, USA) was required, the core surgical success was achieved through complete internal exclusion of both the fistula and the coronary inlet.

**CONCLUSIONS:**

For GCAAs with LV fistulae, the primary surgical objective should be the integrated internal closure of the fistula and the coronary ostium. This approach effectively excludes the aneurysm and prevents recurrence.

## INTRODUCTION

Giant coronary artery aneurysms (GCAAs), defined as exceeding 20 mm in diameter, are rare clinical entities with a prevalence of 0.02%.^1)^ Surgery is indicated to prevent thrombosis or manage obstructive disease; however, the optimal approach is not yet established. We present 2 cases in which standard ligation or resection was technically challenging, requiring an alternative surgical strategy combined with coronary artery bypass grafting (CABG).

## CASE PRESENTATION

**Fig. 1:**
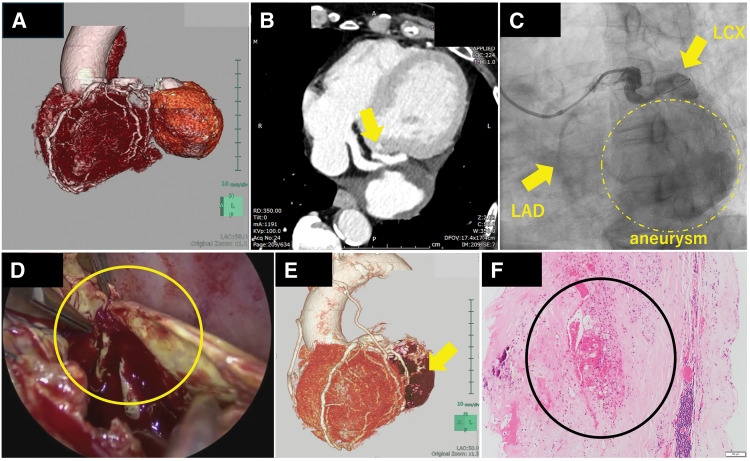
Case 1. (**A**) Preoperative CT revealing a 50 × 70-mm saccular aneurysm in the LCX. (**B**) Preoperative CT (axial view) demonstrating an LV-to-LCX fistula (arrow). (**C**) Coronary angiography showing a giant aneurysm at the distal LCX without clear visualization of the LV-to-LCX fistula. (**D**) Intraoperative internal view showing the neck of the saccular aneurysm. (**E**) Postoperative CT showing the disappearance of the aneurysm (arrow) and a patent CABG. (**F**) Histopathological findings (hematoxylin and eosin stain, original magnification × 100 μm) demonstrating atherosclerotic aneurysmal changes with intimal calcification and foamy macrophage accumulation. CABG, coronary artery bypass grafting; LAD, left anterior descending artery; LCX, left circumflex artery; LV, left ventricular

In Case 1, an 80-year-old man presented with a chief complaint of ankle pain and palpitations. While initial physical examinations were unremarkable, the electrocardiogram (ECG) revealed paroxysmal atrial fibrillation, and laboratory tests showed an elevated C-reactive protein (21.3 mg/dL) and B-type natriuretic peptide (300 pg/mL) that normalized spontaneously. Preoperative CT identified a 50 × 70-mm saccular GCAA in the left circumflex artery (LCX) (**Fig. 1A**) and faintly depicted a left ventricular (LV)-to-LCX fistula (**Fig. 1B**), though PET-CT ruled out active inflammation. Echocardiography indicated reduced cardiac function with an ejection fraction (EF) of 44% and diffuse hypokinesis. While coronary angiography (CAG) confirmed complete occlusion of the right coronary artery, the fistula itself remained unclear (**Fig. 1C**), making it impossible to calculate the shunt magnitude. Due to the anatomical complexity and high rupture risk, surgical treatment was prioritized over a catheter-based approach. Under cardiopulmonary bypass and cardioplegic arrest, the aneurysm ostium was successfully closed directly (**Fig. 1D**), despite severe vessel wall calcification. To ensure distal perfusion and protect the LCX, a left internal thoracic artery-obtuse marginal (LITA-OM) bypass was established, alongside direct fistula closure and aneurysmorrhaphy. Postoperative CT confirmed the disappearance of the aneurysm and a patent CABG (**Fig. 1E**).

**Fig. 2:**
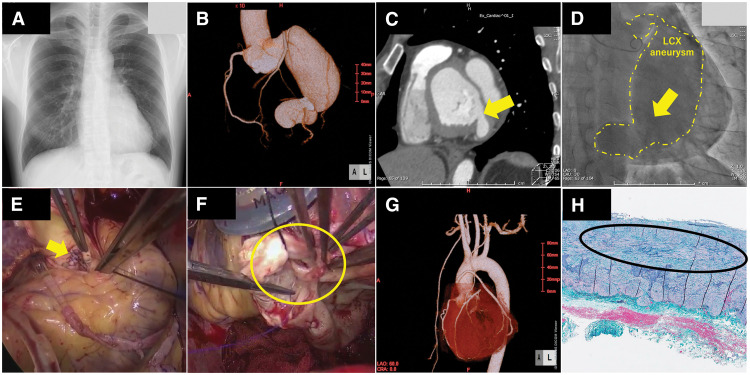
Case 2. (**A**) Chest radiograph showing mediastinal widening. (**B**) Preoperative CT revealing a 50 × 50-mm giant coronary artery aneurysm extending from the LMT to the LCX. (**C**) Preoperative CT (axial view) demonstrating an LV-to-LCX fistula (arrow). (**D**) Coronary angiography showing a giant aneurysm at the LCX with clear visualization of the LV-to-LCX fistula (arrow). (**E**) Surgical closure of the left coronary ostium using a Hemashield patch (arrow) via aortotomy. (**F**) Intraoperative surgeon’s view of the LV-to-LCX fistula. (**G**) Postoperative CT showing resolution of the aneurysm and patent CABG. (**H**) Histopathological findings (Masson’s trichrome stain) revealing cystic medial necrosis characterized by mucoid degeneration of the media and fragmentation of elastic fibers. CABG, coronary artery bypass grafting; Hemashield patch, GETINGE, Göteborg, Sweden; LCX, left circumflex artery; LMT, left main trunk; LV, left ventricular

In Case 2, a 39-year-old man who presented with no primary clinical complaints was referred following an abnormal chest X-ray during a routine health checkup (**Fig. 2A**). ECG indicated sinus rhythm with LV hypertrophy, while echocardiography revealed a reduced EF of 48% and significant LV enlargement (LVDd/Ds = 75/55 mm). Preoperative CT and CAG identified a GCAA stretching from the left coronary ostium to the distal LCX (**Fig. 2B**), accompanied by an LV-to-LCX fistula (**Fig. 2C** and **2D**). The surgical intervention was performed via a median sternotomy under cardiopulmonary bypass and cardioplegic arrest. The procedure involved the direct closure of the left coronary ostium using a Hemashield patch (GETINGE, Göteborg, Sweden) and an LV-to-LCX fistula (**Fig. 2E** and **2F**), combined with CABG to ensure global myocardial revascularization. Although initial weaning from bypass was successful with graft flows (LITA-left anterior descending artery [LAD]: 65 mL/min; pulse index: 1.0; Ao-SV-OM-PL: 52 mL/min, pulse index: 1.9), the patient required prophylactic percutaneous cardiopulmonary support (PCPS) in the ICU due to high-dose catecholamine requirements. A subsequent decline in LV function necessitated the insertion of an Impella (Abiomed, Danvers, MA, USA) for mechanical unloading. Postoperative monitoring showed a peak creatine kinase-MB of 407 U/L; although echocardiography suggested LCX ischemia, postoperative CT confirmed patent CABGs and aneurysm resolution (**Fig. 2G**). Rescue CABG was ultimately abandoned due to the extreme narrowness of the available conduits, likely a long-term sequela of chemotherapy for the patient’s history of acute lymphocytic leukemia (ALL). Intensive management with Impella and heart failure therapy facilitated gradual recovery; the Impella was successfully removed on POD 14. The patient was discharged on POD 30 with an improved EF of 45%. At 3-year follow-up, the patient remains stable without recurrence of the aneurysm or heart failure symptoms.

## DISCUSSION

The propensity of unrecognized coronary aneurysms to develop into obstructive coronary artery disease poses a significant risk for myocardial ischemia and infarction, with GCAAs exhibiting the most unfavorable prognosis.^2)^ Given the rarity of giant aneurysms, therapeutic consensus remains elusive, and robust evidence-based guidelines for selecting between medical and surgical management have yet to be firmly established.

The theoretical advantages of internal orifice closure as a surgical management strategy are as follows: opening the aneurysm provides an excellent surgical field, allowing clear identification of the branch ostium from within the aneurysm. Direct closure thus ensures the complete interruption of blood flow into the aneurysm. Furthermore, this approach is technically straightforward and contributes to reducing cardiac arrest time. While external ligation or resection would certainly be a viable option for a GCAA without fistula formation, given the known concomitance of coronary aneurysms,^3)^ preoperative assessment and screening for vascular anomalies are crucial for surgical planning. We believe that internal orifice closure is the most reliable and logical strategy for cases involving an LV fistula.

Conversely, contemporary reports suggest that transcatheter outcomes—including residual shunt rates and perioperative complications—are comparable to surgical management.^4)^ While transcatheter closure using vascular plugs was technically feasible,^5)^ given the fistula diameters in our cases (4.5 and 8.9 mm), we prioritized surgical intervention. This decision was based on the complex etiology and the presence of GCAAs exceeding 50 mm. Given these factors, surgery remains the superior strategy to definitively eliminate the risks of post-procedural thromboembolism and spontaneous rupture.

The GCAAs reported here were primarily congenital (LV-to-LCX fistula) in etiology. However, histopathological findings of atherosclerosis (**Fig. 1F**) and cystic medial necrosis (**Fig. 2H**) suggest a coexistence of multiple etiologies. Particularly in Case 2, given the patient’s history of childhood ALL, we suspect that secondary vasculitis—though a rare etiology—led to persistent inflammatory destruction of the tunica media.

Regarding the myocardial injury in Case 2, insufficient blood flow in the LCX territory was considered the primary issue, largely attributed to our CABG strategy and the sacrifice of small branches. Preoperatively, despite diffuse hypokinesis, the patient was deemed to have sufficient surgical tolerance, and stable cardioplegia was achieved using combined antegrade and retrograde techniques. Grafts were severely limited by small vessel diameters, potentially due to a history of childhood ALL. Although complete occlusion of the left coronary artery ostium theoretically required independent reconstruction, the limited length of the harvested great saphenous vein (GSV) necessitated a sequential anastomosis. However, given the GSV graft flow of approximately 52 mL/min, a Y-composite graft might have provided better blood flow distribution to the LCX. Additionally, the internal ligation of the OM and PL ostia during coronary aneurysm opening, along with the closure of 2 tiny branches (≤1 mm) unsuitable for CABG, may have further contributed to the myocardial injury.

Finally, effective antithrombotic management is crucial in such complex cases, as emphasized by current clinical guidelines. In Case 1, the patient had been receiving preoperative anticoagulant therapy with a direct oral anticoagulant for paroxysmal atrial fibrillation. Postoperatively, we administered concomitant antiplatelet therapy with aspirin to ensure the long-term patency of the CABG grafts. In Case 2, we initiated a combination of aspirin and warfarin. This dual therapy was specifically prescribed to prevent LV thrombus formation, considering the patient’s reduced cardiac function, which predominantly affected the posterior wall.

## CONCLUSIONS

Surgical treatment of GCAAs demands a highly personalized approach. Clinical success depends on precise preoperative assessment and the ability to adapt surgical techniques to address anatomical challenges.
